# Development and validation of a prediction model for rehospitalization among people with schizophrenia discharged from acute inpatient care

**DOI:** 10.3389/fpsyt.2023.1242918

**Published:** 2023-08-24

**Authors:** Akira Sato, Toshihiro Moriyama, Norio Watanabe, Kazushi Maruo, Toshi A. Furukawa

**Affiliations:** ^1^Department of Health Promotion and Human Behavior, Kyoto University Graduate School of Medicine/School of Public Health, Kyoto, Japan; ^2^Isogaya Hospital, Ichihara, Japan; ^3^Department of Psychiatry, Soseikai General Hospital, Kyoto, Japan; ^4^Department of Biostatistics, Institute of Medicine, University of Tsukuba, Tsukuba, Japan

**Keywords:** schizophrenia spectrum disorder, relapse, prognosis, individualized risk, hospitalization, psychoses

## Abstract

**Objective:**

Relapses and rehospitalization prevent the recovery of individuals with schizophrenia or related psychoses. We aimed to build a model to predict the risk of rehospitalization among people with schizophrenia or related psychoses, including those with multiple episodes.

**Methods:**

This retrospective cohort study included individuals aged 18 years or older, with schizophrenia or related psychoses, and discharged between January 2014 and December 2018 from one of three Japanese psychiatric hospital acute inpatient care ward. We collected nine predictors at the time of recruitment, followed up with the participants for 12 months, and observed whether psychotic relapse had occurred. Next, we applied the Cox regression model and used an elastic net to avoid overfitting. Then, we examined discrimination using bootstrapping, Steyerberg’s method, and “leave-one-hospital-out” cross-validation. We also constructed a bias-corrected calibration plot.

**Results:**

Data from a total of 805 individuals were analyzed. The significant predictors were the number of previous hospitalizations (HR 1.42, 95% CI 1.22–1.64) and the current length of stay in days (HR 1.31, 95% CI 1.04–1.64). In model development for relapse, Harrell’s c-index was 0.59 (95% CI 0.55–0.63). The internal and internal-external validation for rehospitalization showed Harrell’s c-index to be 0.64 (95% CI 0.59–0.69) and 0.66 (95% CI 0.57–0.74), respectively. The calibration plot was found to be adequate.

**Conclusion:**

The model showed moderate discrimination of readmission after discharge. Carefully defining a research question by seeking needs among the population with chronic schizophrenia with multiple episodes may be key to building a useful model.

## 1. Introduction

The prognosis of patients with schizophrenia or related psychoses has not improved over the past few decades. A study by the world health Organization (WHO) showed that during a 15-year follow-up in the 1970s and 1990s, only 38% of those with schizophrenia and 55% of those with other psychoses reached a recovery phase lasting 2 years or longer (1). Similarly, in recent years, only 13.5 to 38% of people with schizophrenia and related psychosis, including those with first-episode psychosis, recovered past 2 years (2, 3). While definitions may vary, poor recovery among people with psychotic disorders indicates a vast unmet need.

One important factor hindering the recovery process is relapse. More than 80% of people with first-episode schizophrenia experience relapse within 5 years of initial recovery (4). Similarly, 63% of such individuals suffer from a relapse within 2 years after their discharge from the hospital, and most of those who relapse are rehospitalized (5). Hospitalization is the most common method for measuring relapse in people with schizophrenia and first-episode psychosis. A systematic review found that 6 of 16 studies used readmission to measure relapse (6), and another showed that 47 of 87 manuscripts reported hospitalization to define relapse (7). Therefore, hospitalization can be used as a quantifiable, easy-to-measure proxy for relapse, which mental health professionals may find easier to discuss with patients and their caregivers.

Multiple prognostic factors may well be related to relapses and rehospitalization. Several prognostic factors, such as adherence problems and expressed emotions, have been identified (7, 8). However, such separately reported prognostic factors do not allow us to predict individual patient prognoses. A prediction model that considers relevant predictors simultaneously and provides personalized risk for each patient is required.

Unfortunately, research on prediction modeling in psychiatry is scarce. A recent systematic review of prediction models in *Psychiatry* included only 89 articles (9). Of these, only seven studied schizophrenia, merely one of which focused on psychotic relapse (10). Moreover, the study used predictors that would not be assessable outside research-oriented academic centers. Another systematic review of prediction models in first-episode psychosis included 13 studies (11). Again, only two of the included studies had an outcome of rehospitalization (12, 13). These articles used covariates that are easily obtained in routine practice; however, they focused on people in the first episode. Therefore, little is known about the personalized risk of relapse and psychiatric rehospitalization in clinical settings, including that in people with schizophrenia after multiple episodes.

To address this issue, we aimed to develop and validate a clinical prediction model that could estimate the risk of relapse, including hospitalization, among people with schizophrenia or related psychoses, including those with multiple episodes, at the time of discharge from acute inpatient care in psychiatric hospitals. Our primary interest is building a model with routinely collected data for people with such illnesses, regardless of their life trajectories.

## 2. Materials and methods

We have previously published our protocol for this study elsewhere (14). We adhered to the Transparent Reporting of a Multivariable Prediction Model for Individual Prognosis or Diagnosis (TRIPOD) for developing and validating our prediction model (Supplementary Table 1) (15). This study was registered in the UMIN-CTR (UMIN000043345) on 20 February 2021. In this section, we briefly summarize our methods.

### 2.1. Study design and source of data

We conducted a retrospective cohort study to obtain datasets for our prediction model. We collected data from three psychiatric hospitals in Japan that differed in their physical venues and care levels. The Chiba Psychiatric Medical Center (CPMC) is a publicly owned tertiary care psychiatric facility that primarily treats psychosis. The Urawa Psychiatric Sanatorium Hospital (UPSH) and Isogaya Hospital (IH) are private secondary care psychiatric hospitals. We collected data only from the acute care ward of the participating hospitals. In Japan, acute care usually provides intensive treatment for people with acutely ill, first-episode, or relapsing psychotic disorders, with an average length of stay of 56.7 days from 2011 onward (16, 17).

### 2.2. Study population

By consecutively reviewing all inpatient records in the three psychiatric hospitals between 2014 and 2018, we recruited people with schizophrenia or related psychoses who were discharged from an acute inpatient ward. We reviewed the medical records of patients admitted to IH and USPH between 1 January 2014, and 31 December 2018. For CPMC, we could access such records between 1 January 2014, and 31 December 2016, excluding those in 2017 and 2018, for administrative reasons. We chose this 5-year period to avoid the influence of concurrent events of the major earthquake and COVID-19 pandemic in 2011 and 2019, respectively.

### 2.3. Eligibility criteria

We included individuals if they:

(1) Were 18 years of age or older;
(2) Had a diagnosis of schizophrenia or related psychoses, including schizotypal disorder, persistent delusional disorders, acute and transient psychotic disorders (ATPD), induced delusional disorder, schizoaffective disorders, other non-organic psychotic disorders, and unspecified non-organic psychosis.
(3) Received inpatient care primarily to treat psychosis; and
(4) Were discharged from an acute inpatient care ward.

The International Classification of Diseases 10th revision (ICD-10) was used for diagnosis (18). If an individual had several hospitalization episodes during the study period, we randomly selected data from one episode.

We excluded individuals who were diagnosed with substance or medication-induced psychotic disorders or psychotic disorders secondary to another medical condition. We excluded patients with a tentative diagnosis of schizophrenia or related psychoses without further evaluation for a definite diagnosis upon discharge. We also excluded individuals who were currently hospitalized for a non-psychotic episode, discharged from a non-acute ward, had an unclear diagnosis, were transferred to another psychiatric/medical facility, or had an immediate plan to return home overseas after discharge. For hospitalization episodes excluded from our study, we recorded age, sex, and reasons for exclusion.

### 2.4. Study outcome

Our primary outcome was time to relapse as a composite outcome defined as the occurrence of any one of the following: (1) rehospitalization, (2) psychiatrist judgment that the patient requires hospitalization, (3) increasing doses of antipsychotics, or (4) suicidal or homicidal ideation or violent behavior resulting in injury to self or another person. All events should occur because of psychotic exacerbation. Our secondary outcome was time to rehospitalization due to psychotic exacerbation within 12 months of discharge. We followed up the discharged individuals by reviewing their outpatient medical records to observe whether they had such outcomes.

### 2.5. Selection of candidate predictors

Before collecting the data, we specified nine predictors based on existing literature and expert opinions. In the literature search, we used a search filter for the concept of prediction (19). The prespecified predictors were age at discharge, sex, number of previous hospitalizations, presence of any hospitalization in the previous year, current length of stay, presence of current substance use disorders, use of long-acting injections at discharge, number of psychosocial interventions during the current hospitalization, and receipt of benefits.

Briefly, previous hospitalizations included any psychiatric admissions in the past, regardless of the type of admission, length of stay, or reasons for hospitalization. We defined hospitalization in the previous year as any hospitalization intended to treat a psychotic episode in the past 12 months before the start of the current hospitalization episode. We counted the number of psychosocial interventions provided during the current hospitalization regardless of the duration of the intervention. Psychosocial interventions include psychoeducational, social skills training, and occupational therapeutic approaches. We excluded any interventions provided to family members because our data sources did not include those records.

We collected the predictors by reviewing inpatient records at the time of their discharge.

### 2.6. Data extraction and data cleaning

We first extracted data on predictors for the included individuals from inpatient records. Relapse data were collected from the outpatient records. All hospitals stored inpatient and outpatient medical records in physically different locations. Two data extractors independently reviewed the medical records of 30 individuals. For data extraction accuracy, we calculated percentage agreements and kappa statistics for binary variables, and an intraclass correlation coefficient (ICC) for continuous variables. We also kept the data extractors blinded to the outcome while extracting baseline data or to the baseline data while judging the outcome, and reported the proportion of data for which this blinding was broken.

For continuous variables of previous hospitalizations, current length of stay, and psychosocial interventions, we identified outliers above the 99th percentiles by creating box plots. We “winsorized” those outliers by shifting very high values to the 99th percentiles. We identified no predictors with a narrow or skewed distribution.

### 2.7. Sample size calculation

To estimate our sample size, we followed the criteria proposed by Riley et al. (20). We calculated the minimum sample size to be 754 to develop our model without overfitting predictor effects.

### 2.8. Model development

We applied a Cox regression model to predict outcomes. We treated both participants who dropped out before the end of the study and those who had no relapse at the 12-month follow-up as censored. From a clinical perspective, we assumed no interaction in our model. We assessed the linearity assumption by performing an overall test and including squared or higher-order polynomials in our model to observe any changes in model performance. Non-linear terms were included in our model if the overall test *p*-value was less than 0.05, or if including non-linear terms improved the performance. To avoid overfitting, we employed an elastic net for penalized estimation of the regression coefficients (21). An elastic net allows for both the selection and penalization of the main effects by introducing two tuning parameters. It also considers the correlations between predictors. Ten-fold cross-validation allowed us to obtain optimal values for the two parameters.

### 2.9. Model performance

We calculated the Brier score for overall accuracy, that is, the extent to which the prediction model could explain the variability in outcomes (22, 23). We estimated Harrell’s C-statistic for discrimination (23, 24), which is the ability of a model to discriminate participants with the outcome from those without the outcome. For a graphical depiction of discrimination, we drew a grouped Kaplan-Meier plot (23). We divided the included individuals into three groups based on tertiles of predicted probabilities of no hospitalization and plotted Kaplan-Meier curves for time-to-observed hospitalization in each of the grouped cohorts. We also examined a calibration plot to determine the agreement between the observed and predicted outcomes (23, 25).

### 2.10. Model validation

We examined both internal and internal-external validity (26). Bootstrap validation with 500 repetitions was performed to assess model reproducibility. We also report the optimism-corrected performance described by Steyerberg (27). Geographical transportability was inspected by “leave-one hospital-out” cross-validation (28). In this internal-external validation, a dataset from one hospital out of the three was excluded to test the performance of the model. A dataset from the remaining two hospitals was used to construct the model. This process was repeated for each of the three hospitals. A bias-corrected calibration plot with 500 bootstraps was constructed for visual inspection of the results.

### 2.11. Sensitivity analyses

We performed sensitivity analyses to observe whether the performance of our model changed. We developed three prediction models for people with schizophrenia only, people with first-episode schizophrenia only, and people aged between 18 and 65 years.

### 2.12. Statistical software

We used R version 4.1.2 for our analyses (29). The packages we employed included *rms* version 6.3-0 (30), *glmnet* version 4.1-4 (31), and *glmnetUtils* version 1.1.8 (32).

### 2.13. Changes from the protocol

From a clinical perspective and owing to the small sample size, we did not perform a statistical analysis for the additivity assumption, as initially planned in our protocol (14). To assess linearity, we introduced squared or higher-order polynomials as predictor variables, in addition to the overall test, as described in the protocol. As we found only four (0.5%) missing values in the baseline data, we did not use multiple imputations, as specified in the protocol. Instead, a complete case analysis was performed. We added a bias-corrected calibration plot. Because our model needed to perform better to be used in clinical practice, we neither performed decision curve analysis nor created a web-based application.

## 3. Results

### 3.1. Participants’ characteristics

Data were collected between January 2021 and June 2022 and analyzed from August to October 2022. Inter-rater reliability showed moderate to excellent agreement for data extraction on predictors and the outcome, and the degree of unblinding during data collection was negligible (Supplementary Tables 2, 3). We did not find any patient overlap between the hospitals.

**FIGURE 1:**
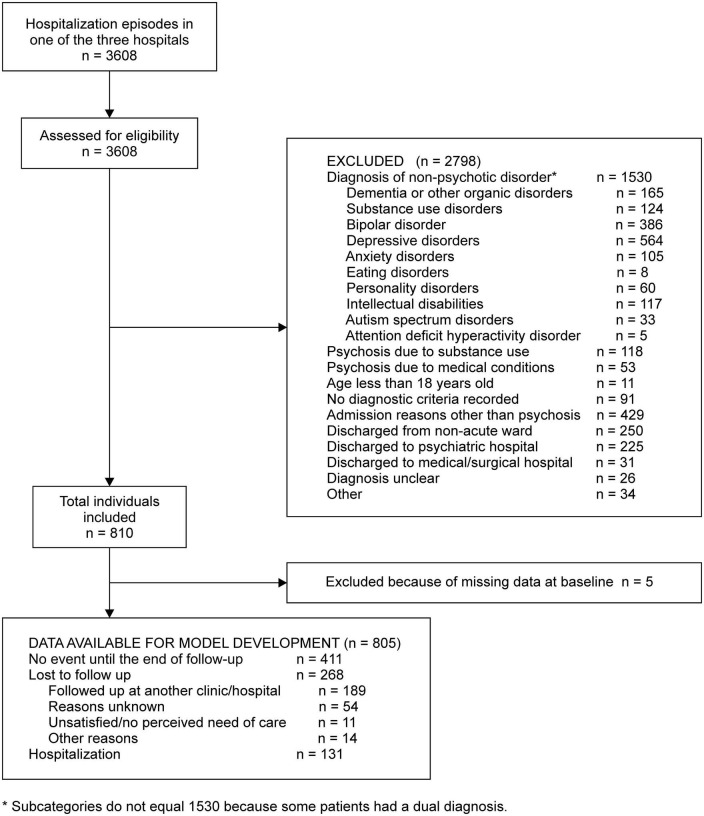
Flow chart of individuals in the model development.

For the medical records between 2014 and 2018, we screened 3,608 hospitalization episodes of discharged individuals. We excluded 2,798 episodes for various reasons with the most frequent reason being diagnosis of non-psychotic disorder (*n* = 1,530), randomly chose one episode for an individual with multiple episodes, and finally included 810 individuals (Figure 1).

Overall, the mean age was 45.1 years (SD 13.8 years), 58.9% were female, 19.0% were hospitalized in the previous year, 14.0% received benefits from their local government, 15.6% were medicated in the form of long-acting injections, and 1.5% were dually diagnosed with substance use disorders (Table 1 and Supplementary Table 4). The median number of previous hospitalizations, current length of stay, and psychosocial intervention sessions during hospitalization were 1 (range, 0–9), 52 (range, 2–205), and 0 (range, 0–34), respectively. Of the cohort, 684 participants (84.0%) were diagnosed with schizophrenia, 57 (7.0%) with ATPD, 48 (5.9%) with schizoaffective disorders, 17 (2.1%) with delusional disorders, and 4 (0.5%) with other diagnoses (Table 1 and Supplementary Table 4).

**TABLE 1:** Baseline characteristics of individuals (*n* = 810).

Characteristic	Number (%)
Age at discharge, mean (SD), y	45.1 (13.8)
Female sex	477 (58.9)
Psychiatric diagnoses (ICD-10 code)	
Schizophrenia (F20)	684 (84.4)
ATPD (F23)	57 (7.04)
Schizoaffective disorder (F25)	48 (5.93)
Delusional disorder (F22)	17 (2.10)
Others (F21, F24, F28, F29)	4 (0.50)
Receipt of benefits	114 (14.1)
Number of previous hospitalizations, median (range)	1 (0 to 15)
Hospitalization in the previous year	153 (19.0)
Current length of stay in days, median (range)	52 (2 to 207)
Use of long-acting injections at discharge	126 (15.6)
Current substance use disorder	12 (1.48)
Number of psychosocial interventions, median (range)	0 (0 to 34)

ATPD, acute and transient psychotic disorders.

We excluded five individuals because of missing data. Of the remaining 805 individuals, 411 (51.1%) had no hospitalization episodes until the end of follow-up, 268 (33.3%) were lost to follow-up, and 131 (16.3%) were hospitalized (Figure 1). After inspecting the non-linear terms, we included nine predictors in our final model, as specified in our protocol (Supplementary Table 5). The significant predictors of hospitalization within 12 months of discharge were the number of previous hospitalizations (HR 1.42, 95% CI 1.22–1.64) and the current length of stay in days (HR 1.31, 95% CI 1.04–1.64) (Table 2).

**TABLE 2:** Association between pre-specified predictors and hospitalization from the ridge regression in the complete-case analysis (*n* = 805).

Variable	Hazard ratio (95% CI)
Age at discharge	0.87 (0.69–1.12)
Female sex	0.89 (0.63–1.27)
Receipt of benefits	1.20 (0.75–1.90)
Number of previous hospitalizations	1.42 (1.22–1.64)
Current length of stay	1.31 (1.04–1.64)
Current substance use disorders	0.70 (0.10–5.15)
Number of psychosocial interventions	0.95 (0.87–1.03)
Use of long-acting injections at discharge	0.63 (0.34–1.04)
Hospitalization in the previous year	1.23 (0.80–1.89)

### 3.2. Model development and validation

**FIGURE 2:**
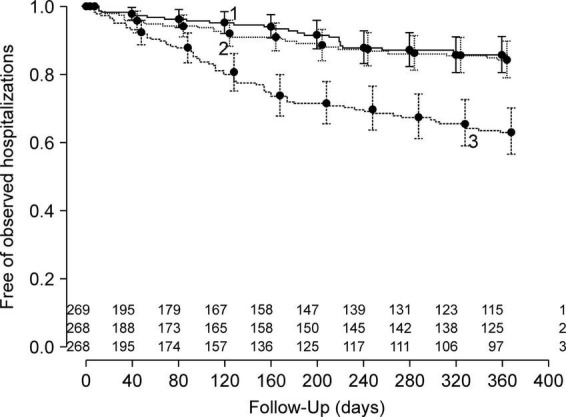
Fractions of individuals free of observed hospitalizations in three groups according to tertiles of predicted probabilities of no hospitalization in the model development.

**FIGURE 3:**
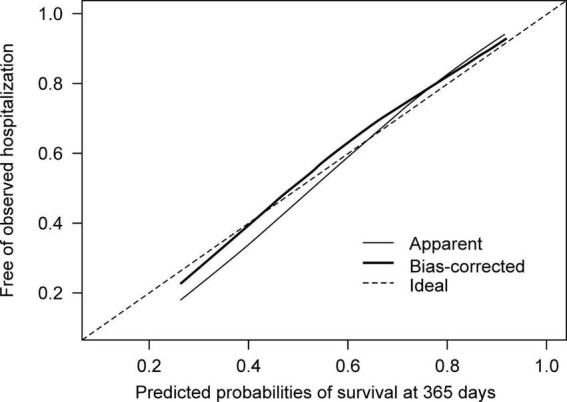
Calibration plots for the predicted probabilities of no hospitalization against proportions of individuals free of observed hospitalization.

In developing a model for relapse broadly defined as a composite outcome, we found that Harrell’s c-index was 0.59 (95% CI 0.55–0.63) and did not proceed to further analysis. Hereafter, we describe the findings from the model of the secondary outcome of rehospitalization. In the model development, the overall accuracy in the Brier scores at fixed time points was 0.07, 0.12, and 0.16 on day 90, 180, and 360 after discharge, respectively. The Kaplan-Meier plot showed the proportion of individuals free of observed hospitalization in the three groups based on tertiles of predicted survival (i.e., no hospitalization) (Figure 2). In groups 1 and 2, 25 of 269 and 32 of 268 patients had hospitalization episodes, respectively. In contrast, 74 of 268 patients had hospitalization episodes in group 3, the group with the worst predicted survival. For regularization of coefficients in the nine predictors to avoid overfitting, the elastic net selected ridge regression over least absolute shrinkage and selection operator (LASSO) regression. In model validation, our model showed moderate discrimination. The internally validated Harrell’s c-index from Steyerberg’s optimism-corrected measure and the bootstrapping were 0.64 (95% CI 0.59–0.69) and 0.64 (95% CI 0.61–0.71), respectively. For the internal-external validation, the average of the three c-indices from the “leave-one hospital-out” cross-validation was 0.66 (95% CI 0.57–0.74). The bias-corrected calibration plot using bootstrapping indicated an adequate calibration of the predicted probabilities of no hospitalization against the observed proportions of non-hospitalization (Figure 3).

### 3.3. Sensitivity analysis

The sensitivity analysis included three different models: people with schizophrenia only (i.e., excluding those with other psychoses), people with the first episode of hospitalization, and those aged between 18 and 65 (i.e., excluding elderly patients). In model development, Harrell’s c-index for each of the three prediction models showed results similar to those of the primary analyses (Supplementary Table 6).

## 4. Discussion

In this retrospective cohort study, we described the development and validation of a prediction model for readmission after hospital discharge in individuals with schizophrenia or related psychoses who had a history of no, single, or multiple hospitalizations. To use the model in everyday practice, we focused on nine routinely collected predictors at the time of discharge. Our final model showed moderate discrimination for rehospitalization, and the internally and internal-externally validated Harrell’s c-index were 0.64 and 0.66, respectively.

When we built a model with relapse as a composite outcome instead of rehospitalization alone, the model’s discrimination ability was close to no better at prediction of relapse compared to random chance (Harrel’s c-index, 0.59). The difficulty in observing the components of our composite outcome in paper-based medical records may account for this poor discrimination. We found it difficult to follow relapse occurrences because handwritten documents were sometimes difficult to read, poorly organized, or even damaged. We also suspect that it was difficult to observe the broadly defined relapse outcome, because some physicians did not record a relapse other than hospitalization. For example, physicians may not record why they increased antipsychotic doses or how many days a patient used antipsychotics prescribed as needed. In addition, they may not record police or ambulance involvement, as such involvement is not strictly a psychiatric issue. However, we did not have such problems with collecting predictors and hospitalization because they were simply numbers or recorded as a single word. The relatively low agreement in inter-rater reliability for relapse compared to other variables may support this speculation (Cohen’s kappa 0.71, Supplementary Table 2).

That said, the discrimination ability of our model may not increase even if we do not overlook any components of the composite outcome. We believe that all the components were measured subjectively and that many factors influence subjective judgment; for instance, a physician may increase the antipsychotic dose when a patient seems agitated. However, agitation may or may not result from psychotic exacerbations. Physicians may not have enough time to distinguish these differences but may increase the dosage if it is due to psychosis. In addition, we could not include factors that occurred during follow-up, which could have influenced the prognosis of individuals after discharge. These factors may include adherence to medication at home, psychological distress from a row with family members, and job loss during the index hospitalization. One or more of these factors may interact with a patient’s life after discharge and influence one or more components of the composite outcome.

When we built the model for rehospitalization, its moderate discrimination (Harrel’s c-index 0.66) was comparable to that of previous studies that included similar outcomes or populations. A model predicting readmission after hospital discharge in individuals with first-episode psychosis had a c-index of 0.66 (95% CI not shown) in the model validation (12). Another model using LASSO, which predicted the occurrence of non-organic psychotic disorders, 70% of which were schizophrenia, following ATPD, showed similar discrimination at 1-year follow-up (area under the curve [AUC] 0.678, 95% CI not shown) (33). Furthermore, a model predicting transition to psychosis in individuals at clinical high risk reported a c-index of 0.665 (95% CI 0.627–0.682) (34). However, the discrimination ability of our model, among others, may hinder its use in clinical settings. Models that forecast the outcome of changes in psychotic conditions may require future research to improve their performance for use in clinical practice.

As for predictors, the hazard ratios of the two predictors were statistically significant, while the other seven showed otherwise. However, among those with negative findings, the upper confidence limits of the number of psychosocial interventions and the use of long-acting injections at discharge, for example, were close to one: Had we had a larger sample size, they may have shown significant effects. However, non-significant findings for each predictor do not necessarily exclude themselves from a model (35). Prediction models produce estimation rather than hypothesis testing. Negative, non-significant results do not imply a zero effect. We pre-specified predictors based on the literature. Including all the pre-defined predictors in our model still did not achieve clinically useful predictive power.

On the other hand, prediction models for the behavior of people with severe mental illnesses may be promising. A study using the same data source presented different prediction models for people with severe mental illness, 63% of which were schizophrenia. One model predicting violent offences within 1 year showed good discrimination ability (c-index 0.89, 95% CI 0.85–0.93) (36). Another model for suicide within 1 year reported the measure of discrimination to a lesser extent (c-index 0.71, 95% CI 0.66–0.75) (37). However, we should notice that when the former model was externally validated in another dataset, with a slightly different outcome, the AUC decreased to 0.67 (95% CI 0.61–0.73) (38). We consider that the generalizability of a model can still be challenging.

Our study has several limitations. First, we were not able to include some important prognostic factors. For example, the emotion expressed by a patient’s family is an important predictor of relapse (39). We did not include adherence to antipsychotics; although we had identified this factor as a candidate predictor in our literature search, we were obliged to discard it in the present study because our data sources did not record the variable. Another limitation is the possibility of underestimating the number of rehospitalizations. In total, 189 individuals were transferred to another mental health facility (Figure 1). Many of these mental health facilities do not provide inpatient care; when transferred patients experience psychotic relapse and need hospitalization, they may or may not be referred back to our hospital. When not referred back, these patients’ relapses and rehospitalizations were not accounted for in our dataset.

By contrast, the strengths of this study lie in our endeavor to demonstrate its robustness. We performed a systematic literature search to pre-specify predictors and precisely defined outcomes, registered this study beforehand in the clinical trial registration system, and published our protocol in a peer-reviewed journal. The study period was carefully selected to avoid confounding due to concurrent events. We extracted data from the three different hospitals to examine their geographical transportability. We assessed the inter-rater reliability to ensure that the data extraction was trustworthy.

## 5. Conclusion

Here, we present a prediction model designed not only for first-episode admission, but also for the population of schizophrenia with multiple episodes. Our model, with routinely collected data from three psychiatric hospitals, showed moderate discrimination of psychotic readmission after hospital discharge. We speculate that depending on the complex nature of an outcome, it may be challenging to forecast such an outcome within a year, regardless of the predictors we choose. Carefully defining a research question by seeking needs among the population with chronic schizophrenia with multiple episodes, for example, using qualitative interviews, may be key to building a useful model.

## Data availability statement

The datasets presented in this article are not readily available because individual privacy could be compromised. Requests to access the datasets should be directed to AS, asatomatsu@gmail.com.

## Ethics statement

The studies involving humans were approved by the Ethics Committee of Kyoto University Graduate School and Faculty of Medicine (no. R2710-2) and the Ethics Committee of CPMC and IH. The Ethics Committee of Kyoto University Graduate School and Faculty of Medicine also approved the study on behalf of UPSH as UPSH did not hold such a committee. The studies were conducted in accordance with the local legislation and institutional requirements. The Ethics Committee/institutional review board waived the requirement of written informed consent for participation from the participants or the participants’ legal guardians/next of kin because the investigator would be unable to obtain consent for many participants because they had moved or lost to follow-up.

## Author contributions

AS, NW, and TAF contributed to the conception of this study and designed the overall framework. TAF was the principal investigator of this study protocol. AS wrote the manuscript in consultation with KM, NW, TAF, and TM. AS and TM independently collected data for assessing the reliability of data extraction. All authors read and approved the final manuscript.
