# Supplementary material for: Development and validation of a prediction model for rehospitalization among people with schizophrenia discharged from acute inpatient care
Source: Front Psychiatry. 2023 Aug 24;14:1242918. doi: 10.3389/fpsyt.2023.1242918 (PMC10483840; doi:10.3389/fpsyt.2023.1242918)
Supplement: Supplementary file 1 [file Table_1.docx]

Supplementary Material

Development and validation of a prediction model for rehospitalization among people with schizophrenia discharged from acute inpatient care

Akira Sato*, Toshihiro Moriyama , Norio Watanabe, Kazushi Maruo, Toshi A. Furukawa

*** Correspondence:** Corresponding Author: asatomatsu@gmail.com

# Supplementary Tables

**Supplementary Table 1.** TRIPOD Checklist: Prediction Model Development and Validation.

| **Section/Topic** | **Item** |  | **Checklist Item** | **Page** |
| --- | --- | --- | --- | --- |
| **Title and abstract** | | | | |
| Title | 1 | D;V | Identify the study as developing and/or validating a multivariable prediction model, the target population, and the outcome to be predicted. | 1 |
| Abstract | 2 | D;V | Provide a summary of objectives, study design, setting, participants, sample size, predictors, outcome, statistical analysis, results, and conclusions. | 1 |
| **Introduction** | | | | |
| Background and objectives | 3a | D;V | Explain the medical context (including whether diagnostic or prognostic) and rationale for developing or validating the multivariable prediction model, including references to existing models. | 2 |
|  | 3b | D;V | Specify the objectives, including whether the study describes the development or validation of the model or both. | 2 |
| **Methods** | | | | |
| Source of data | 4a | D;V | Describe the study design or source of data (e.g., randomized trial, cohort, or registry data), separately for the development and validation data sets, if applicable. | 2 |
|  | 4b | D;V | Specify the key study dates, including start of accrual; end of accrual; and, if applicable, end of follow-up. | 2 |
| Participants | 5a | D;V | Specify key elements of the study setting (e.g., primary care, secondary care, general population) including number and location of centres. | 2 |
|  | 5b | D;V | Describe eligibility criteria for participants. | 2, 3 |
|  | 5c | D;V | Give details of treatments received, if relevant. | n/a |
| Outcome | 6a | D;V | Clearly define the outcome that is predicted by the prediction model, including how and when assessed. | 3 |
|  | 6b | D;V | Report any actions to blind assessment of the outcome to be predicted. | 3 |
| Predictors | 7a | D;V | Clearly define all predictors used in developing or validating the multivariable prediction model, including how and when they were measured. | 3 |
|  | 7b | D;V | Report any actions to blind assessment of predictors for the outcome and other predictors. | 3 |
| Sample size | 8 | D;V | Explain how the study size was arrived at. | 3 |
| Missing data | 9 | D;V | Describe how missing data were handled (e.g., complete-case analysis, single imputation, multiple imputation) with details of any imputation method. | 4 |
| Statistical analysis methods | 10a | D | Describe how predictors were handled in the analyses. | 3 |
|  | 10b | D | Specify type of model, all model-building procedures (including any predictor selection), and method for internal validation. | 3, 4 |
|  | 10c | V | For validation, describe how the predictions were calculated. | 3 |
|  | 10d | D;V | Specify all measures used to assess model performance and, if relevant, to compare multiple models. | 3, 4 |
|  | 10e | V | Describe any model updating (e.g., recalibration) arising from the validation, if done. | n/a |
| Risk groups | 11 | D;V | Provide details on how risk groups were created, if done. | n/a |
| Development vs. validation | 12 | V | For validation, identify any differences from the development data in setting, eligibility criteria, outcome, and predictors. | 3, 4 |
| **Results** | | | | |
| Participants | 13a | D;V | Describe the flow of participants through the study, including the number of participants with and without the outcome and, if applicable, a summary of the follow-up time. A diagram may be helpful. | 4, Fig 1 |
|  | 13b | D;V | Describe the characteristics of the participants (basic demographics, clinical features, available predictors), including the number of participants with missing data for predictors and outcome. | 4, Table 1 |
|  | 13c | V | For validation, show a comparison with the development data of the distribution of important variables (demographics, predictors and outcome). | Supplementary Table 4 |
| Model development | 14a | D | Specify the number of participants and outcome events in each analysis. | 4, Fig 1 |
|  | 14b | D | If done, report the unadjusted association between each candidate predictor and outcome. | n/a |
| Model specification | 15a | D | Present the full prediction model to allow predictions for individuals (i.e., all regression coefficients, and model intercept or baseline survival at a given time point). | Supplementary Table 5 |
|  | 15b | D | Explain how to the use the prediction model. | n/a |
| Model performance | 16 | D;V | Report performance measures (with CIs) for the prediction model. | 4 |
| Model-updating | 17 | V | If done, report the results from any model updating (i.e., model specification, model performance). | n/a |
| **Discussion** | | | | |
| Limitations | 18 | D;V | Discuss any limitations of the study (such as nonrepresentative sample, few events per predictor, missing data). | 7 |
| Interpretation | 19a | V | For validation, discuss the results with reference to performance in the development data, and any other validation data. | 6 |
|  | 19b | D;V | Give an overall interpretation of the results, considering objectives, limitations, results from similar studies, and other relevant evidence. | 5-7 |
| Implications | 20 | D;V | Discuss the potential clinical use of the model and implications for future research. | 6, 7 |
| **Other information** | | | | |
| Supplementary information | 21 | D;V | Provide information about the availability of supplementary resources, such as study protocol, Web calculator, and data sets. | 2, 8 |
| Funding | 22 | D;V | Give the source of funding and the role of the funders for the present study. | See below |

Funding: No funding support was received for this study. Reference: Sato, A., Watanabe, N., Maruo, K. et al. Psychotic relapse in people with schizophrenia within 12 months of discharge from acute inpatient care: protocol for development and validation of a prediction model based on a retrospective cohort study in three psychiatric hospitals in Japan. Diagn Progn Res 6, 20 (2022). https://doi.org/10.1186/s41512-022-00134-w

**Supplementary Table 2.** Inter-rater reliability of predictors and relapse for the 30 consecutive participants.

|  | ICC | Cohen's Kappa | Percentage agreement |
| --- | --- | --- | --- |
| Eligibility criteria |  | 0.814 | 93.3 |
| Age at discharge | 0.999 |  |  |
| Sex |  | 0.930 | 96.7 |
| Receipt of benefit |  | 0.902 | 96.7 |
| Total number of past hospitalizations | 0.847 |  |  |
| Current length of stay | 0.986 |  |  |
| Current SUD use |  | 0 | 96.7 |
| Total psychosocial sessions | 1.000 |  |  |
| Current LAI use |  | 0.701 | 86.7 |
| Hospitalization in the previous year |  | 0.933 | 96.7 |
| Relapse |  | 0.714 | 86.7 |

ICC, intraclass correlation coefficient. LAI, long-acting injections. SUD, substance use disorders.

**Supplementary Table 3.** Proportions of unblinded data during data collection (n=810).

|  | Number (%) |
| --- | --- |
| Unblinded at baseline (i.e., the data extractor knew the outcome) | 67 (8.3) |
| Unblinded during the follow-up (i.e., the data extractor knew the condition of at least one of nine predictors) | 38 (4.7) |

**Supplementary Table 4.** Baseline characteristics of individuals in each hospital*.

| Characteristic | All hospitals (n = 810) (%) | CPMC (n = 370)(%) | IH (n = 161) (%) | UPSH (n = 279) (%) |
| --- | --- | --- | --- | --- |
| Age at discharge, mean (SD), y | 45.1 (13.8) | 41.9 (12.3) | 47.3 (14.8) | 48.1 (14.3) |
| Female sex | 477 (58.9) | 222 (60.0) | 90 (55.9) | 165 (59.1) |
| Psychiatric diagnoses (ICD-10 code) |  |  |  |  |
| Schizophrenia (F20) | 684 (84.4) | 316 (85.4) | 127 (78.9) | 241 (86.4) |
| ATPD (F23) | 57 (7.04) | 27 (7.3) | 10 (6.2) | 20 (7.2) |
| Schizoaffective disorder (F25) | 48 (5.93) | 21 (5.7) | 15 (9.3) | 12 (4.3) |
| Delusional disorder (F22) | 17 (2.10) | 6 (1.6) | 9 (5.6) | 2 (0.7) |
| Others (F21, F24, F28, F29) | 4 (0.50) | 0 (0.0) | 0 (0.0) | 4 (1.4) |
| Receipt of benefits | 114 (14.1) | 33 (8.9) | 22 (13.7) | 59 (21.2) |
| Number of previous hospitalizations, median (range) | 1 (0 to 15) | 1 (0 to 15) | 1 (0 to 15) | 1 (0 to 15) |
| Hospitalization in the previous year | 153 (19.0) | 49 (13.2) | 31 (19.3) | 73 (26.5) |
| Current length of stay in days, median (range) | 52 (2 to 207) | 45 (2 to 207) | 65 (5 to 207) | 61 (6 to 207) |
| Use of long-acting injections at discharge | 126 (15.6) | 56 (15.1) | 59 (36.7) | 11 (3.9) |
| Current substance use disorder | 12 (1.48) | 5 (1.35) | 2 (1.24) | 5 (1.80) |
| Number of psychosocial interventions, median (range) | 0 (0 to 34) | 0 (0 to 19) | 0 (0 to 34) | 4 (0 to 34) |

* For continuous variables of previous hospitalizations, current length of stay, and psychosocial interventions, we "winsorized" those outliers by shifting very high values to the 99th percentiles. ATPD, acute and transient psychotic disorders. CPMC, Chiba Psychiatric Medical Center. IH, Isogaya Hospital. UPSH, Urawa Psychiatric Sanatorium Hospital.

**Supplementary Table 5.** Presenting the final ridge model, including the baseline survival, for a specific time point*.

|  | Beta coefficient |
| --- | --- |
| Age at discharge | -0.007158979 |
| Sex | -0.109747286 |
| Number of previous hospitalizations | 0.113617134 |
| Presence of any hospitalization in the previous year | 0.210998992 |
| Current length of stay | 0.005070876 |
| Presence of current substance use disorders | -0.340880953 |
| Use of long-acting injections at discharge | -0.453464154 |
| Number of psychosocial interventions during the current hospitalization | -0.017031005 |
| Receipt of benefits | 0.175664749 |

* S_0_(365) = 0.7817099 (365-day baseline survival).

**Supplementary Table 6.** Sensitivity analysis comparing three different models for hospitalization with the original model in the model development.

|  | n | Events | Harrell’s c-index (95% CI) |
| --- | --- | --- | --- |
| Original model | 805 | 131 | 0.667 (0.618–0.716) |
| Model including individuals aged < 65 | 734 | 122 | 0.669 (0.618–0.720) |
| Model including individuals with first episode of hospitalization only | 315 | 26 | 0.662 (0.568–0.755) |
| Model including individuals with schizophrenia only | 679 | 114 | 0.650 (0.597–0.704) |
